# Draft Genome Sequence of the Yeast *Pseudozyma antarctica* Type Strain JCM10317, a Producer of the Glycolipid Biosurfactants, Mannosylerythritol Lipids

**DOI:** 10.1128/genomeA.00878-14

**Published:** 2014-09-25

**Authors:** Azusa Saika, Hideaki Koike, Tomoyuki Hori, Tokuma Fukuoka, Shun Sato, Hiroshi Habe, Dai Kitamoto, Tomotake Morita

**Affiliations:** a Research Institute for Innovation in Sustainable Chemistry, National Institute of Advanced Industrial Science and Technology (AIST), Tsukuba, Ibaraki, Japan; b Bioproduction Research Institute, National Institute of Advanced Industrial Science and Technology (AIST), Tsukuba, Ibaraki, Japan; c Research Institute for Environmental Management Technology, National Institute of Advanced Industrial Science and Technology (AIST), Tsukuba, Ibaraki, Japan

## Abstract

The basidiomycetous yeast *Pseudozyma antarctica* is known as a producer of industrial enzymes and the extracellular glycolipids, mannosylerythritol lipids. Here, we report the draft genome sequence of the type strain JCM10317. The draft genome assembly has a size of 18.1 Mb and a G+C content of 60.9%, and it consists of 197 scaffolds.

## GENOME ANNOUNCEMENT

*Pseudozyma antarctica* is a basidiomycetous yeast belonging to the class *Ustilaginomycetes*, which includes the plant pathogen, *Ustilago maydis* (1, 2). The species is known to produce attractive enzymes, which are used in a wide range of industrial fields (3, 4). In recent years, the type strain has also received considerable attention as a producer of the glycolipid biosurfactants known as mannosylerythritol lipids (MELs) (5). The type strain of *P. antarctica* is thus a promising organism for the industrial production of several attractive materials; however, little about this strain is known. Here, we report the draft genome sequence of *P. antarctica* JCM10317 (CBS 214.83), the type strain isolated from lake sediment at Antarctica, formerly *Sporobolomyces antarctica* (2).

The paired-end DNA library (insert size: ~500 bp) was prepared using a NEBNext Ultra DNA Library Prep Kit for Illumina (New England BioLabs, Ipswich, MA, USA), and sequencing was performed using the Illumina platform (MiSeq). Sequence data totaling 11,086,474 paired-end reads, each 250 bp in length, were generated. Additionally, a mate-paired library (insert size: ~3200 bp) was prepared using a Nextera Mate Pair Sample Prep Kit (Illumina, San Diego, CA, USA), and sequencing resulted in 10,198,878 mate-paired reads. The Allpaths-LG assembler (6) was used to generate 276 contigs with 65.5× and 69.2× genome coverage from the paired-end and mate paired libraries, respectively. The contigs derived from the two libraries were assembled into 197 scaffolds. Accordingly, the draft genome size of strain JCM10317 was 18.1 Mb (G+C content: 60.9%). The length of the longest scaffold was 2,397 kb, and the *N*_50_ length was 701,213 kb with eight scaffolds.

Using the database of *U. maydis* UM521 (MIPS *U. maydis* Database, http://mips.helmholtz-muenchen.de/genre/proj/ustilago) as references, 6,845 protein-coding genes were automatically predicted using the AUGUSTUS program (7), and 5,901 (86.2%) and 5,877 (85.9%) of the genes were found to be homologous to proteins in the *P. antarctica* T-34 (accession no. DF196767 to DF196793) (8) and RefSeq protein database (9) of the National Center for Biotechnology Information (BLASTp e-value: 1e−10; aligned region: ≥70% of the reference sequence; sequence identity: ≥25%), respectively. In addition, 57.8% of the genes were also tentatively assigned protein function(s) based on EuKaryotic Orthologous Groups (KOG) classification by searching against the eggNOG database (10).

The gene responsible for lipase B (CALB), which is a major industrial enzyme, was located on scaffold 7. The five genes encoding erythritol/mannose transferase (*emt1*), acyl transferase (*mac1* and *mac2*), putative transporter (*mmf1*), and acetyl transferase (*mat1*), which are responsible for MEL biosynthesis, were conserved on scaffold 31 as a gene cluster identical to those of the other MEL producers, *P. antarctica* T-34 (11), and shared 94.8%, 91.1%, 86.8%, 93.4%, and 92.4% identity. The genome data of the *P. antarctica* JCM10317 will facilitate understanding of its genomic characteristics and lead to further utilization of the species in a range of industries.

### Nucleotide sequence accession numbers.

The nucleotide sequence of the *P. antarctica* JCM10317 genome has been deposited in DDBJ/EMBL/GenBank under the accession numbers BBIZ01000001 to BBIZ01000276 (as WGS entries) and DF830068 to DF830264 (as 197 entries).
